# Seasonal fluctuation of oribatid mite communities in forest microhabitats

**DOI:** 10.7717/peerj.4863

**Published:** 2018-06-04

**Authors:** Katja Wehner, Michael Heethoff, Adrian Brückner

**Affiliations:** Ecological Networks, Technische Universität Darmstadt, Darmstadt, Germany

**Keywords:** Oribatida, Forest microhabitats, Environmental niche, Temperature, Relative humidity, Network analysis, Litter, Dead wood, Moss, Acari

## Abstract

Oribatid mites are abundant and diverse decomposers in almost all terrestrial microhabitats, especially in temperate forests. Although their functional importance in the decomposition system in these forests has been investigated, spatio-temporal patterns of oribatid mite communities inhabiting different microhabitats have largely been neglected. Therefore, we (i) investigated seasonal fluctuation (monthly over one year) in oribatid-mite community structure and specificity to three microhabitats (moss, dead wood and litter) and (ii) analyzed the influence of air temperature and overall air humidity on seasonal community changes. In total, 57,398 adult oribatid mite individuals were collected. Total abundance, species richness and diversity differed among microhabitats. Seasonal changes were most pronounced in moss and least in litter. While overall air humidity had no influence on species distribution and community changes, air temperature positively influenced species richness and diversity, again most pronounced in moss. The calculated environmental temperature occurrence niche showed that 35% of adult oribatid mite species occurred at higher air temperatures. Furthermore, interaction/bipartite networks were more generalized—i.e., species were more equally distributed among moss, dead wood and litter—when ambient air temperatures were higher. This pattern is probably due to the dispersal ability of adult oribatid mites, i.e., species enter a dispersal mode only at higher air temperatures.

## Introduction

The stability of belowground systems is driven by biotic (community composition, biodiversity or competition) and abiotic (precipitation, air temperature or soil chemistry/nutrients) factors (Seastedt, 1984; Wardle, 2006; Eisenhauer et al., 2012). Especially nutrient cycling and decomposition are important processes for the persistence of forests and their biota (Peterson & Luxton, 1982; Smith et al., 2014). While functional roles and contributions of soil fauna—for example protozoans, nematodes, collembolans, oribatid mites and earthworms—have been studied to some extent (Bayoumi, 1978; Eisenhauer, 2010; Maaßet al., 2015; Soong & Nielsen, 2016), mechanisms driving soil fauna diversity remain enigmatic (Eisenhauer et al., 2017).

Oribatid mites (Oribatida, Arachnida) are among the most diverse and abundant animal decomposers. The approximately 16,000 described species (Schatz et al., 2011) are collectively ubiquitous, yet individual species are unevenly distributed in all major forests and grasslands, and also in aquatic habitats all over the world (Wallwork, 1983; Schatz, 2005; Schatz & Behan-Pelletier, 2008). In temperate forests, oribatid mite species tend to inhabit different microhabitat patches and therefore their communities are unequally distributed among mineral soil, litter, mosses, lichens, dead wood or tree bark (Aoki, 1967; Arroyo, Kenny & Bolger, 2013; Wehner et al., 2016). This specificity may be caused by differences in microhabitat structure (e.g., small vs wide pores, continuous (litter) vs. insular (tree bark, moss); Nielsen et al., 2008), microclimatic conditions (e.g., moisture, exposure; Nielsen et al., 2010), spatial resource heterogeneity (Nielsen et al., 2010) or biotic interactions (e.g., predation; Hammer, 1972; Gao et al., 2014). While litter and forest soils are continuous and relatively stable habitats (Maraun & Scheu, 2000), the most unique oribatid mite fauna is found on trees (Lindo & Winchester, 2006; Skubala & Gurgul, 2011; Lindo & Winchester, 2012).

Oribatid mite communities in forest soil systems are affected by small- and large-scale environmental factors (Nielsen et al., 2008; Nielsen et al., 2010; Corral-Hernández, Balanzategui & Iturrondobeitia, 2016). Due to their small size and restricted dispersal ability, soil communities and especially oribatid mites therein mainly respond to local rather than regional factors (Erdmann, Scheu & Maraun, 2012; Bolger et al., 2014; Corral-Hernández, Balanzategui & Iturrondobeitia, 2016). Spatio-temporal patterns of oribatid communities inhabiting different forest microhabitats have largely been neglected in the past (Gergócs et al., 2011).

In general, most adult oribatid mite species tend to prefer distinct microhabitats (e.g., litter, moss patches, dead wood, lichens, grassy sods, bark of trees) if conditions are favourable (Wehner et al., 2016). For example, Wehner et al. (2016) reported that the microhabitat ‘litter’ provides the most stable ecological conditions and seems to function as habitat for litter specialists (e.g., *Hypochthonius rufulus*; Habitat-Hypothesis) and “refuge” for generalists during unfavourable conditions (e.g., *Chamobates cuspidatus*, *Carabodes* spp.; “Connector-Hypothesis”). Nevertheless, in more patchy microhabitats such as mosses, dead wood or lichens, overall abundance and diversity of oribatid mite communities can exceed those of litter (Skubala, 2008; Wehner et al., 2016). The interaction of factors driving the distribution patterns of oribatid mite species within and among different microhabitats are complex. Besides high niche dimensionality and resource portioning (Mitchell, 1979; Wardle, 2002) as well as differential rates of activity (Chase & Leibold, 2003), environmental variables and local species interactions are of great importance (Caruso, Toarmina & Migliorini, 2012; Maaßet al., 2015). We further assume local weather and seasonality of community structure to influence distribution pattern of oribatid mite species.

Seasonality of oribatid mites have mainly been investigated in soil and litter habitats in Middle European forests (Harding, 1969; Hammer, 1972; Mitchell, 1977; Schenker, 1984). However, since oribatid mite groups differ in their ecology (i.e., small, fast reproducing Oppiidae vs large, slowly reproducing Nothrina), results of seasonality studies are not always consistent (Schenker, 1984) Generally, seasonality changes seem to be small, but adult oribatid mites tend to show one to two peaks of high abundances (Harding, 1969; Hammer, 1972; Luxton, 1972) caused by their reproductive cycle (i.e., egg deposition in spring, large numbers of nymphs in May and June, adults in late summer; Hammer, 1972).

In this study, we aimed to (i) investigate seasonal fluctuation in oribatid-mite community structure in, and specificity to three different forest microhabitats (moss, dead wood and litter) and (ii) understand the influence of weather (air temperature and relative air humidity) on community changes. Therefore, we investigated abundance, richness and diversity in these micro-communities monthly over one year and analyzed community structure using microhabitat-specific bipartite networks. Furthermore, we calculated an environmental occurrence niche model (Chisté et al., 2016; Mangels et al., 2017) for each oribatid mite species to understand and explain their distributions among microhabitats and changes in network structure. We hypothesized that (i) the microhabitats (moss, dead wood, litter) differ in their community structure (abundance, species richness and Shannon diversity of oribatid mites) and that (ii) seasonality of species distribution and specialization differs according to community composition and microhabitat structure. We further assumed that (iii) seasonality is driven by weather conditions (air temperature, air humidity) in all three microhabitats.

## Materials and Methods

### Sample locations

In 2016, samples of moss, dead wood and litter were taken in the State Forest 2043 A in Mörfelden-Walldorf (N49°58′30.8424/E8°33′1.0332; 96 m a.s.l.) about 15 km north of Darmstadt, Hesse, Germany. The oak-mixed forest is moderate subcontinental and mesotrophic, the surface is flat and covered with sand. The main tree population includes pine (*Pinus sylvestris*), birch (*Petula pendula*), oak (*Quercus robur*), beech (*Fagus sylvatica*) and spruce (*Picea abies*) with an approximate age of 67 years. The sub-canopy layer consists of 35 year old beech, oak, sorbus (*Sorbus* sp.), birch, pine, willow (*Salix* sp.), cherry (*Prunus* sp.), maple (*Acer* sp.), elm (*Ulmus* sp.), lime (*Tilia* sp.) and common hornbeam (*Carpinus betulus*), the shrub-layer comprises blackberry (*Rubus section*), wild garlic (*Allium ursinum*), and ivy (*Hedera helix*); data provided by the forest management plan of the Forestry office Groß-Gerau.

### Sampling procedure

Samples of moss (including *Mnium undulatum*, *Polytrichum* cf *formosum*, *Amblystegium varium*, *Brachythecium* sp., *Dicranella* sp. and *Eurhynchium* sp., depending on sampling date), prostrate dead wood of different decaying stages and litter material were taken haphazardly in a sampling area (30 m × 30 m) at the beginning of each month (January to December) in 2016 (ten replicates each, 30 samples per month, 360 samples in total). Litter was removed by hand (about 20 cm × 20 cm) including the organic material on the ground surface, pieces of dead wood (about 10 cm × 10 cm) were snapped from prostrate dead wood on the forest floor, and moss patches (including a mixture of different species) were only partly removed to reach a sample size of at least 5 g wet weight. Samples were collected in plastic bags and transferred to the laboratory.

Microarthropods were extracted for 48 h using a modified Kempson heat extractor (Kempson, Llyod & Ghelardi, 1963) and stored in 75% ethanol. Samples were weighed after extraction and dry weights of samples were used to standardize the number of individuals as Ind/kg dry weight following (Skubala, 2016) (dry weights can be found in Table S1). Adult oribatid mites were determined to species, genus or family level under a microscope using the key of Weigmann (2006). Taxonomic classification was adapted from Weigmann (2006), Norton & Behan-Pelletier (2009), Schatz et al. (2011), and Subías (2014).

### Weather parameters

For monitoring the parameters air temperature and relative air humidity, one data logger was placed on each of the four edges of the 30 m × 30 m sampling area; two at a height of 1.5 m on trees and two about 2 cm above the litter layer. A plastic roof covered each logger. Relative air humidity and air temperature were logged every 3,600 s. Loggers were checked during the sampling dates. For statistical analyses, data of air temperature and air humidity were pooled for all four loggers for the last five days before the specific sampling date and used for all three microhabitats.

### Statistical analysis

We analyzed our data using three different approaches. (i) We compared community parameters (total abundance, species richness, effective Shannon diversity), their seasonal changes and air temperature/humidity effects among microhabitats. (ii) We analyzed microhabitat specificity and influences of weather on oribatid mite-habitat distribution network structure over one year. (iii) We analyzed the environmental niches of all species.

Oribatid mite abundance (Ind/kg dry weight), species richness (N, mean number of species per sample) and effective Shannon diversity (*e*^*H*^; Jost, 2006) were statistically analyzed as response variables, while microhabitat (always fitted first) and month or weather parameters, i.e., air temperature or relative air humidity (always fitted second) were fixed as explanatory variables. For the time analyses we additionally included month as random effect to account for the consecutive nature of the month data. For the month-based time analyses of abundance and species richness we used generalized linear mixed effects models (GLMM) with a negative-binomial error distribution and log as link-function, while we fitted a linear mixed effect model (LMM) for the effective Shannon diversity. For the weather parameter analyses we used generalized linear models (GLMs). The GLMs for oribatid mite abundance was fitted with a quasi-Poisson error distribution and log as link-function, while the GLMs for oribatid mite species richness was fitted with a negative-binomial error distribution and log as link-function. Effective Shannon diversity (*e*^*H*^) of oribatid mites was analyzed with Gaussian error distribution GLMs and identity as link-function. Prior to the GLM/GLMM/LMM analyses we tested the error distribution of the response variables (abundance, richness, diversity) using goodness-of-fit tests.

Network analysis has previously been used to investigate the specialization (i.e., the complementary distribution) of oribatid mite species to different forest microhabitats (Wehner et al., 2016). Here, we used network analyses to map changes in complementary specialization ${H}_{2}^{{^{\prime}}}$ (${H}_{2}^{{^{\prime}}}$ symbolizes a specialization index at network level; Blüthgen, Menzel & Blüthgen, 2006) and network structure of oribatid mite communities to their microhabitats (moss, dead wood, litter) over the year. The ${H}_{2}^{{^{\prime}}}$ of the monthly networks were compared against a null model with fixed marginal totals using the original counted data (for the *RxC randomization* algorithm see Patefield, 1981). We used the observed ${H}_{2}^{{^{\prime}}}$ and compared it against 10,000 randomized networks with the same marginal totals, resulting in a null ${H}_{2}^{{^{\prime}}}$ for every month, which was compared to the observed one (for details see Blüthgen, Menzel & Blüthgen, 2006; Wehner et al., 2016). To test how strong the networks of consecutive months were correlated with each other we used a Mantel test analogous approach; we first standardized each network (based on the original counted data) to link temperature (i.e., the deviation of observed-neutral interaction strength; see Junker, Höcherl & Blüthgen (2010) for details) and subsequently used a permutative Mantel test based model (*N* = 1,000 permutations) as well as a correlation test, to obtain the p-statistics and correlation coefficient r (mean ± SD) for each consecutive month pair. Afterwards, we used GLMs to test the influence of weather parameters (air temperature, relative air humidity) on the complementary specialization ${H}_{2}^{{^{\prime}}}$ (Gaussian error distribution, link-function= indentity).

We further calculated the standardized Kullback–Leibler divergence *d*′ (Blüthgen, Menzel & Blüthgen, 2006) for each microhabitat per month; *d*′ quantifies the selective occurrence of species across the microhabitats. Accordingly, a higher *d*′ denotes a higher exclusiveness (i.e., higher specialization) of species occurring in a certain microhabitat. Since *d*′ and the pairwise ${H}_{2}^{{^{\prime}}}$ distance (see (Blüthgen, Menzel & Blüthgen, 2006) for details) of the microhabitats were highly correlated (Pearson’s product-moment correlation: *r* = 0.91; *P* < 0.001), ${H}_{2}^{{^{\prime}}}$ can be used as a decent indicator to compare the microhabitat specificity within each month. Consequently, we calculated the pairwise ${H}_{2}^{{^{\prime}}}$ distance of dead wood, litter and moss of each month and tested their differences (again using a RxC randomization on the original counted data with *N* = 1,000 permutations). The influence of air temperature and relative air humidity on measured *d*′ values of each month was analyzed using one-way multivariate analyses of variance (Wilk’s lambda MANOVA), due to the non-independence of *d*′ within each month. Subsequently, we used univariate protected ANOVAs (Scheiner & Gurevitch, 2001) to check the significance of the individual microhabitats.

To further characterize the change of network parameters/architecture, we calculated the environmental niche for each oribatid mite species, as the abundance-weighted means (*μ*_*i*_) of air temperature (*ϑ*) or relative air humidity (RH). The *μ*_*i*_ (Eq. (1)) of a species i is the sum product of proportion p of individuals of species i found in month m in relation to its total abundance, and the measured air temperature or relative air humidity in month *m* (for details see Chisté et al., 2016).

(1) \begin{eqnarray*}{\mu }_{i}=\sum _{m=1}^{12}{p}_{m,i}\ast {\vartheta }_{m} \text{or} R{H}_{m}\end{eqnarray*}

Hence, *μ*_*i*_ denotes the mean air temperature or relative air humidity of occurrence of a certain oribatid mite species, while the calculated standard deviation of *μ*_*i*_ represents the niche breadth of a species. To statistically analyze the observed *μ*_*i*_ we compared them to a null model, which assumes a random distribution of species across all months. The null model calculated *μ*_perm_ for 10,000 iterations, and compared these results with the observed *μ*_*i*_for each species to estimate a *p*-value for the deviation between observed *μ*_*i*_ and the permutated *μ*_perm_ values (see Chisté et al., 2016; Mangels et al., 2017 for details). The environmental occurrence niche represents the parameter (air temperature and air humidity) width where a species could be found in the three microhabitats over the year.

All statistical analyses were performed with R 3.3.2—“Sincere Pumpkin Patch” (R Development Core Team, 2014), using the packages “bipartite” (Dormann, Gruber & Fründ, 2008), “lme4” (Bates et al., 2015), “nlme” (Pinheiro et al., 2016), “MASS” (Venables & Ripley, 2002), “DHARMa” (Hartig, 2017), “car” (Fox & Weisberg, 2011) and “SDMTools” (Van der Wal et al., 2014).

## Results

In total, we collected 57,398 adult oribatid mite individuals, representing 57 species; two genera (*Carabodes*, *Tectocepheus*) and three families (Brachychthoniidae, Phthiracaridae and Suctobelbidae) were not determined to the species level. In general, Brachypylina were most abundant (50 taxa), followed by Nothrina (five taxa), Enarthronota (four taxa) and Mixonomata (three taxa; Table 1; see Table S2 for detailed overview). We found no species from the infraorders Paleosomata or Parhyposomata.

As expected, air temperatures were highest from April to October (between 11 and 18 °C) and lowest in winter months (between 0.4 °C in December and 7.4 °C in February; Table 2). Relative air humidity (Table 2) was lowest in May (58%), followed by January (79%), April (88%) and September (89%), highest values were measured in March and November (100%), yet there was no clear humidity trend over the year. Air temperature and air humidity were not significantly correlated (Spearman’s rank: *ρ*_*s*_ =  − 0.14, *P* = 0.66).

**Figure 1:**
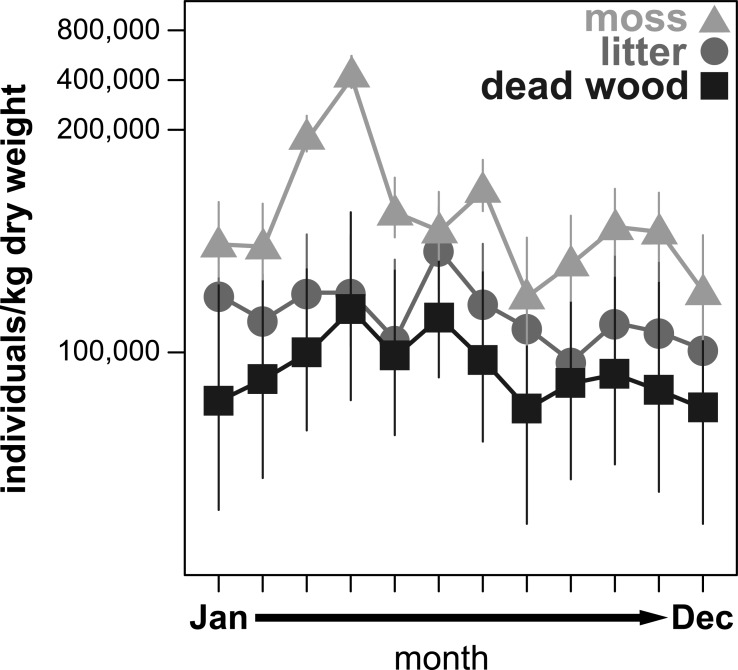
Seasonal fluctuations of oribatid mite abundances (individuals/kg dry weight) in the microhabitats moss, litter and dead wood from January to December 2016. Symbols denote means, while error bar stands are the standard error (SE). Colors correspond to the figure legend.

**Figure 2:**
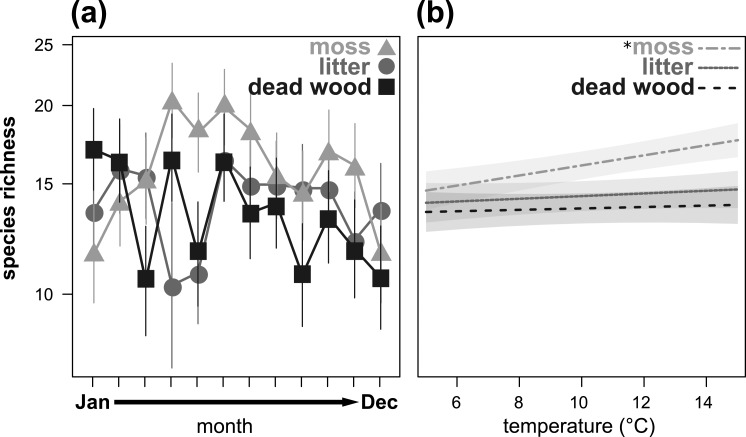
Seasonal fluctuations of oribatid mite species richness (*n* species) in the microhabitats moss, litter and dead wood from January to December 2016 (A) and the influence of temperature (in °C) on species richness in moss, litter and dead wood (B). Symbols denote means, while error bar stands are the standard error (SE). Colors correspond to the figure legend. Grey areas are the 95% confidential intervals. * = significant (*P* < 0.05) trend of temperature in the marked microhabitat.

**Figure 3:**
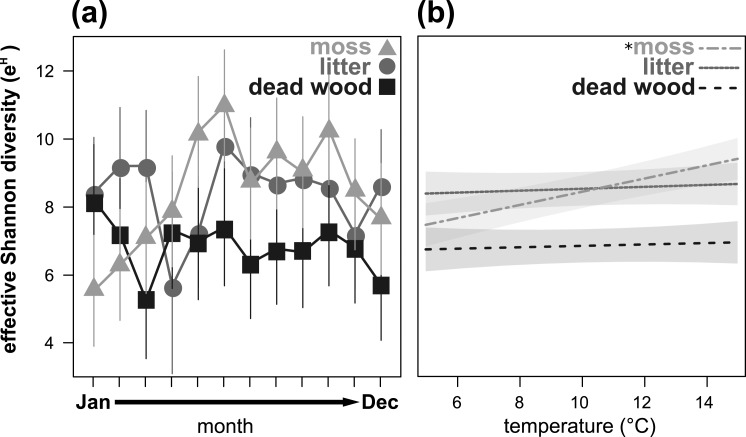
Seasonal fluctuations of the effective Shannon diversity (*e*^*H*^) in the microhabitats moss, litter and dead wood from January to December 2016 (A) and the influence of temperature (in °C) on the effective Shannon diversity in moss, litter and dead wood (B). Symbols denote means, while error bar stands are the standard error (SE). Colors correspond to the figure legend. Grey areas are the 95% confidential intervals. *, significant (*P* < 0.05) trend of temperature in the marked microhabitat.

All community parameters (total abundance, species richness and effective Shannon diversity) significantly fluctuated over the year (Figs. 1–3). Total abundance of adult oribatid mites (Ind/kg dry weight) showed a microhabitat specific change over the year (Table 3, Fig. 1). Total abundance was highest in moss and lowest in dead wood. Seasonal changes were most pronounced in moss (having highest abundance from March to July) and least in litter (Fig. 1). Both air temperature and air humidity had no significant influence on total abundance (Table 3).

Species richness also showed a significant microhabitat specific trend over the year (Table 3, Fig. 2A), being highest in moss from April to July and in October/November. In January and February, as well as in December, species richness was highest in dead wood and litter. Air temperature significantly influenced species richness in moss, but had no effect on dead wood and litter (Table 3, Fig. 2B). Again, air humidity had no significant effect (Table 3).

**Table 1:** Total and mean abundances (Ind/kg dry weight) of oribatid mites from January to December 2016.

	Moss	dw	Litter	Moss	dw	Litter
	Ind/kg	Ind/kg	Ind/kg	mean ± SD (CV%)	mean ± SD (CV%)	mean ± SD (CV%)
**Enarthronota**						
Brachychthoniidae spp. (unidentified)	134,471	10,619	2,682	11,206 ± 18,823 (168)	885 ± 830 (94)	224 ± 241 (108)
*Cosmochthonius lanatus* (Michael, 1885)	0	40	0	0 ± 0 (0)	3 ± 11 (332)	0 ± 0 (0)
*Eniochthonius minutissimus* (Berlese, 1903)	421,610	164,559	48,119	35,134 ± 57,649 (164)	13,713 ± 8,554 (62)	4,010 ± 2,248 (56)
*Hypochthonius rufulus* (Koch, 1835)	68,605	8,057	17,728	5,717 ± 7,950 (139)	671 ± 488 (73)	1,477 ± 1,799 (122)
**Total**	**624,686**	**183,275**	**68,530**	**52,057 ± 73,048 (140)**	**15,273 ± 8,760 (57)**	**5,711 ± 3,650 (64)**
**Mixonomata**						
*Microtritia minima* (Berlese, 1904)	3,179	25,504	3,398	265 ± 837 (316)	2,125 ± 2,344 (110)	283 ± 515 (182)
Phthiracaridae spp. (unidentified)	536,127	298,994	75,466	44,677 ± 52,880 (118)	24,916 ± 24,875 (100)	6,289 ± 2,894 (46)
*Rhysotritia duplicata* (Grandjean, 1953)	0	125	0	0 ± 0 (0)	10 ± 35 (332)	0 ± 0 (0)
**Total**	**539,305**	**324,624**	**78,864**	**44,942 ± 52,888 (118)**	**27,052 ± 24,184 (89)**	**6,572 ± 2,795 (43)**
**Nothrina**						
*Camisia spinifer* (Koch, 1835)	2,100	0	0	175 ± 342 (195)	0 ± 0 (0)	0 ± 0 (0)
*Nanhermannia nana* (Nicolet, 1855)	1,984	766	1,814	165 ± 291 (176)	64 ± 83 (129)	151 ± 110 (73)
*Nothrus palustris* (Koch, 1839)	2,533	1,385	5,723	211 ± 410 (194)	115 ± 89 (77)	477 ± 532 (112)
*Nothrus silvestris* (Koch, 1839)	42,847	2,647	10,125	3,571 ± 5,146 (144)	221 ± 235 (107)	844 ± 518 (61)
*Platynothrus peltifer* (Koch, 1839)	114,433	19,862	112,682	9,536 ± 11,462 (120)	1,655 ± 1,168 (71)	9,390 ± 5,262 (56)
**Total**	**163,897**	**24,661**	**130,344**	**13,658 ± 11,581 (85)**	**2,055 ± 1,348 (66)**	**10,862 ± 5,498 (51)**
**Brachypylina**						
*Adoristes ovatus* (Koch, 1839)	248,111	21,508	66,936	20,676 ± 17,656 (85)	1,792 ± 1,795 (100)	5,578 ± 5,119 (92)
*Achipteria coleoptrata* (Linné, 1758)	37,850	8,255	34,428	3,154 ± 5,799 (184)	688 ± 1,099 (160)	2,869 ± 2,166 (75)
*Achipteria nitens* (Nicolet, 1855)	38,587	6,592	24,612	3,216 ± 5,733 (178)	549 ± 1,002 (182)	2,051 ± 3,500 (171)
*Astegistes pilosus* (Koch, 1840)	2,033	2,102	664	169 ± 324 (191)	175 ± 212 (121)	55 ± 83 (150)
*Autogneta longilamellata* (Michael, 1885)	152,172	76,903	91	12,681 ± 38,115 (301)	6,409 ± 10,702 (167)	8 ± 25 (332)
*Banksinoma lanceata* (Michael, 1885)	41,800	27,520	0	3,483 ± 5,782 (166)	2,293 ± 2,598 (113)	0 ± 0 (0)
*Berniella sigma* (Strenzke, 1951)	15,921	371	362	1,327 ± 2,323 (175)	31 ± 57 (185)	30 ± 60 (198)
*Carabodes* spp. (Koch, 1835)	272,400	56,007	24,443	22,700 ± 23,853 (105)	4,667 ± 3,258 (70)	2,037 ± 1,559 (77)
*Cepheus cepheiformis* (Nicolet, 1855)	90,862	5,968	17,417	7,572 ± 4,503 (59)	497 ± 877 (176)	1,451 ± 972 (67)
*Ceratoppia bipilis* (Hermann, 1904)	19,922	1,098	340	1,660 ± 2,186 (132)	92 ± 165 (181)	28 ± 35 (122)
*Ceratozetes cf gracilis* (Michael, 1884)	2,000	0	214	167 ± 373 (224)	0 ± 0 (0)	18 ± 59 (332)
*Chamobates borealis* (Trägardh, 1902)	19,600	250	0	1,633 ± 5,417 (332)	21 ± 69 (332)	0 ± 0 (0)
*Chamobates cuspidatus* (Michael, 1884)	509,359	19,014	46,862	42,447 ± 53,113 (125)	1,585 ± 1,828 (115)	3,905 ± 3,897 (100)
*Chamobates subglobulus* (Oudemanns, 1900)	1,200	643	141	100 ± 277 (277)	54 ± 165 (308)	12 ± 31 (265)
*Cultroribula bicultrata* (Berlese, 1905)	2,127	2,066	204	177 ± 373 (210)	172 ± 339 (197)	17 ± 26 (150)
*Cymberemaeus cymba* (Nicolet, 1855)	20,800	1,407	2,528	1,733 ± 3,683 (212)	117 ± 117 (100)	211 ± 564 (268)
*Damaeus gracilipes* (Kulczynski, 1902)	72,100	647	118	6,008 ± 19,777 (329)	54 ± 179 (332)	10 ± 33 (332)
*Damaeus onustus* (Koch, 1841)	211,229	17,771	19,894	17,602 ± 21,471 (122)	1,481 ± 1,654 (112)	1,658 ± 2,162 (130)
*Dissorhina ornata* (Oudemans, 1900)	28,634	885	453	2,386 ± 2,446 (103)	74 ± 116 (157)	38 ± 54 (144)
*Dometorina plantivaga* (Berlese, 1895)	18,035	375	48	1,503 ± 3,581 (238)	31 ± 74 (238)	4 ± 13 (332)
*Eupelops plicatus* (Koch, 1836)	149,786	5,868	5,851	12,482 ± 7,587 (61)	489 ± 646 (132)	488 ± 390 (80)
*Euzetes globulus* (Nicolet, 1855)	20,084	7,859	15,828	1,674 ± 1,231 (74)	655 ± 478 (73)	1,319 ± 890 (67)
*Galumna lanceata* (Oudemans, 1900)	0	2,092	498	0 ± 0 (0)	174 ± 342 (196)	42 ± 43 (104)
*Hermannia gibba* (Koch, 1839)	1024,849	156,063	14,109	85,404 ± 115,232 (135)	13,005 ± 12,401 (95)	1,176 ± 910 (77)
*Liacarus coracinus* (Koch, 1841)	68,472	51,217	635	5,706 ± 7,236 (127)	4,268 ± 3,052 (72)	53 ± 52 (98)
*Liacarus subterraneus* (Koch, 1844)	0	40	0	0 ± 0 (0)	3 ± 11 (332)	0 ± 0 (0)
*Licneremaeus licnophorus* (Michael, 1882)	30,788	267	0	2,566 ± 7,213 (281)	22 ± 57 (255)	0 ± 0 (0)
*Liebstadia longior* (Berlese, 1908)	370	40	0	31 ± 92 (297)	3 ± 11 (332)	0 ± 0 (0)
*Liebstadia similis* (Michael, 1888)	1,833	220	531	153 ± 300 (196)	18 ± 43 (235)	44 ± 64 (144)
*Metabelba pulverosa* Strenzke, 1953	81,211	3,944	42,291	6,768 ± 8,554 (126)	329 ± 278 (85)	3,524 ± 3,471 (99)
*Medioppia subpectinata* (Oudemans, 1900)	1,063,064	250,431	120,330	88,589 ± 184,503 (208)	20,869 ± 29,447 (141)	10,028 ± 11,682 (117)
*Microppia minus* (Paoli, 1908)	24,083	15,823	1,181	2,007 ± 3,292 (164)	1,319 ± 2,298 (174)	98 ± 273 (278)
*Multioppia laniseta* (Moritz, 1966)	111,423	70,901	50,008	9,285 ± 14,883 (160)	5,908 ± 10,100 (171)	4,167 ± 7,988 (192)
*Oppia denticulata* (G. & R. Canestrini, 1882)	4,600	3,765	0	383 ± 904 (236)	314 ± 703 (224)	0 ± 0 (0)
*Oppiella falcata* (Paoli, 1908)	218,719	8,480	108	18,227 ± 52,295 (287)	707 ± 1,396 (198)	9 ± 20 (224)
*Oppiella nova* (Oudemans, 1902)	191,072	282,102	3,105	15,923 ± 26,750 (168)	23,508 ± 9,275 (39)	259 ± 199 (77)
*Oribatella quadricornuta* (Michael, 1880)	12,430	564	642	1,036 ± 1,143 (110)	47 ± 84 (180)	54 ± 82 (153)
*Oribatula tibialis* (Nicolet, 1855)	3,033	5,915	144	253 ± 369 (146)	493 ± 835 (169)	12 ± 27 (224)
*Pantelozetes paolii* (Oudemans, 1913)	0	222	1,957	0 ± 0 (0)	19 ± 61 (332)	163 ± 294 (180)
*Peloptulus phaenotus* (Koch, 1844)	200	0	0	17 ± 55 (332)	0 ± 0 (0)	0 ± 0 (0)
*Poroliodes farinosus* (Koch, 1840)	40,972	3,001	790	3,414 ± 3,939 (115)	250 ± 262 (105)	66 ± 82 (125)
*Punctoribates punctum* (Koch, 1839)	200	125	0	17 ± 55 (332)	10 ± 35 (332)	0 ± 0 (0)
*Quadroppia quadricarinata* (Michael, 1885)	1,139,077	225,312	7,097	94,923 ± 109,540 (115)	18,776 ± 16,167 (86)	591 ± 399 (67)
*Scapheremaeus palustris* (Sellnick, 1924)	1,000	0	0	83 ± 276 (332)	0 ± 0 (0)	0 ± 0 (0)
*Scheloribates laevigatus* (Koch, 1835)	47,006	4,973	1,620	3,917 ± 1,1001 (281)	414 ± 797 (192)	135 ± 326 (241)
Suctobelbidae spp. (unidentified)	494,987	66,672	39,185	41,249 ± 37,556 (91)	5,556 ± 4,647 (84)	3,265 ± 2,639 (81)
*Tectocepheus* spp. (Berlese 1813)	2,399,116	450,593	28,453	199,926 ± 262,582 (131)	37,549 ± 31,549 (84)	2,371 ± 2,314 (98)
*Xenillus clypeator* Robineau-Desvoidy, 1839	14,969	3,515	1,054	1,247 ± 1,209 (97)	293 ± 414 (141)	88 ± 87 (99)
*Zygoribatula exilis* (Nicolet, 1855)	445,671	29,091	1,531	37,139 ± 51,415 (138)	2,424 ± 2,977 (123)	128 ± 228 (178)
**Total**	**9,393,759**	**1,898,478**	**576,700**	**782,813 ± 910,058 (116)**	**158,206 ± 107634 (68)**	**48,058 ± 25,044 (52)**

**Notes.**

dw dead wood SD standard deviation CV coefficient of variation

**Table 2:** Weather (temperature, humidity) and network parameters from January to December 2016.

	Temp °C	RH %	${H}_{2}^{{^{\prime}}}$	*d*′ habitat		
				Dead wood	Litter	Moss
January	2.1 ± 1.4	79 ± 16	0.35	0.39	0.36	0.30
February	7.4 ± 2.8	92 ± 12	0.40	0.32	0.49	0.41
March	3.6 ± 1.0	100 ± 17	0.24	0.22	0.38	0.15
April	11.1 ± 3.2	88 ± 19	0.19	0.17	0.31	0.08
May	12.1 ± 3.3	58 ± 21	0.36	0.26	0.54	0.28
June	16.7 ± 1.2	99 ± 10	0.23	0.23	0.33	0.16
July	17.0 ± 1.4	94 ± 9	0.10	0.10	0.16	0.07
August	16.0 ± 2.8	93 ± 12	0.19	0.18	0.27	0.14
September	18.1 ± 1.3	89 ± 12	0.22	0.14	0.41	0.13
October	13.1 ± 1.9	97 ± 10	0.25	0.30	0.23	0.19
November	5.1 ± 1.3	100 ± 7	0.32	0.35	0.34	0.27
December	0.4 ± 2.4	97 ± 10	0.49	0.51	0.42	0.51

**Notes.**

Temp temperature in °C RH % relative humidity; values are means ± standard deviation *H*_2_′ complementary specialization of a bipartite network *d*′ standardized Kullback–Leibler divergence which quantifies the selective occurrence of species across the microhabitats

**Table 3:** Statistical values for total abundances, species richness and effective Shannon diversity. Significant values are given in bold.

	*df*	*F*∕*χ*^2^	*P*
**A - Total abundance**			
**GLMM with negative binomial distribution**			
Habitat	2	35.90	<0.001
Month	11	25.80	0.007
Habitat × Month	22	56.69	**<0.001**
Residual	313		
**GLM with quasi-Poisson distribution**			
Habitat	1	19.04	**<0.001**
Temperature	2	0.08	0.770
Habitat × Temperature	2	0.21	0.812
Residual	343		
**GLM with quasi-Poisson distribution**			
Habitat	1	64.49	**<0.001**
Moisture	2	0.01	0.906
Habitat × Moisture	2	0.09	0.910
Residual	343		
**B - Species richness**			
**GLMM with negative binomial distribution**			
Habitat	2	11.47	0.003
Month	11	50.73	<0.001
Habitat × Month	22	76.57	**<0.001**
Residual	313		
**GLM with negative binomial distribution**			
Habitat	1	0.12	0.943
Temperature	2	0.32	0.573
Habitat × Temperature	2	7.54	**0.023**
Residual	343		
**GLM with negative binomial distribution**			
Habitat	1	6.71	**0.035**
Moisture	2	0.42	0.516
Habitat × Moisture	2	5.95	0.051
Residual	343		
**C - Effective Shannon diversity**			
**LMM**			
Habitat	2	3.75	0.024
Month	11	0.56	0.862
Habitat × Month	22	2.11	**0.003**
Residual	313		
**GLM with Gaussian distribution**			
Habitat	1	4.03	0.019
Temperature	2	0.25	0.614
Habitat × Temperature	2	5.82	**0.003**
Residual	343		
**GLM with Gaussian distribution**			
Habitat	1	0.49	0.391
Moisture	2	1.29	0.257
Habitat × Moisture	2	1.69	0.187
Residual	343		

**Notes.**

*df* degrees of freedom GLMM generalized linear mixed effects models GLM generalized linear models LMM linear mixed effect model

Effective Shannon diversity (*e*^*H*^) showed a significant microhabitat-specific trend over the year (Table 3, Fig. 3A). While *e*^*H*^ was highest from May to November in moss, it remained constant (but lower) in dead wood. In litter, *e*^*H*^ was lowest in April and November, but exceeded *e*^*H*^ of moss in winter (December to March). Again, air temperature had a significant influence on *e*^*H*^ depending on the microhabitat: in moss *e*^*H*^ increased with air temperature, while there were no trends in dead wood and litter (Table 3, Fig. 3B). Air humidity did not influence *e*^*H*^ of oribatid mites in any microhabitat (Table 3).

**Figure 4:**
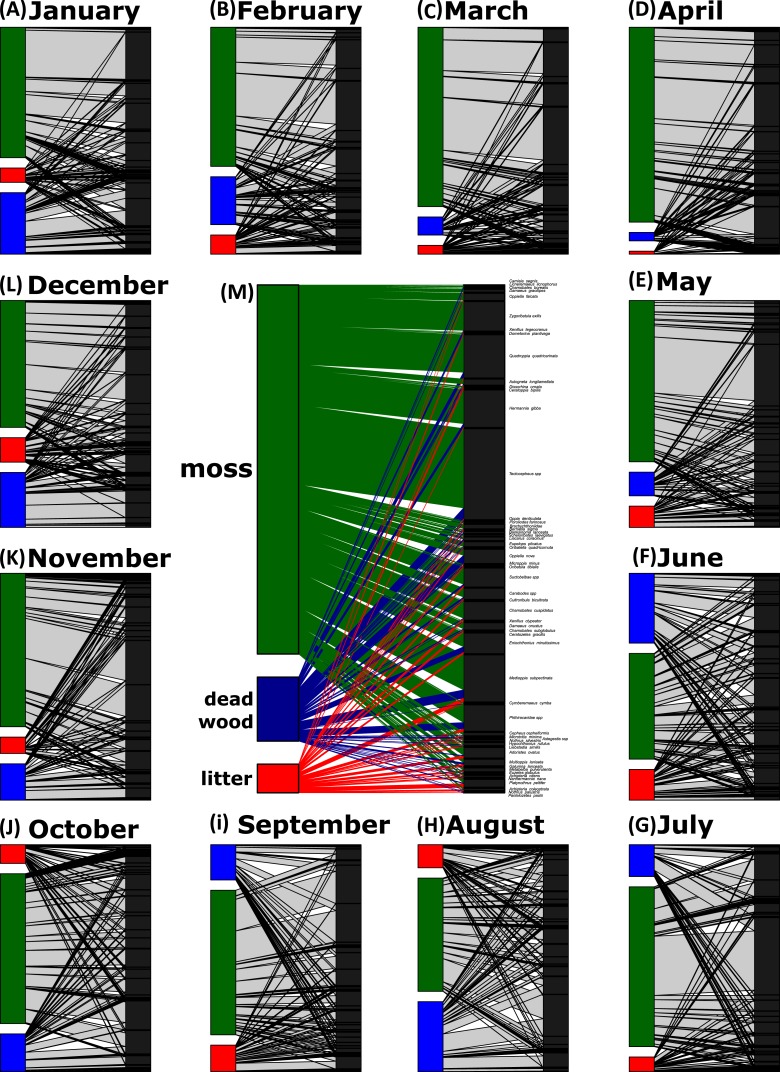
Oribatid mite—microhabitat networks over the year. Networks are based on the number of individuals per kilogram dry weight. The central network is a pooled network of all samples (M), while the other networks show the distribution of oribatid mites across microhabitats on a monthly base (A–L). The width of the bars denotes the number of individuals/kg dry weight in a certain microhabitat (left part of the bipartite graph) or the number of individuals/kg dry weight per species (right part of the bipartite graph). Width of the connecting lines indicate the species abundance. Moss is colored in green, dead wood in blue and litter in red.

**Figure 5:**
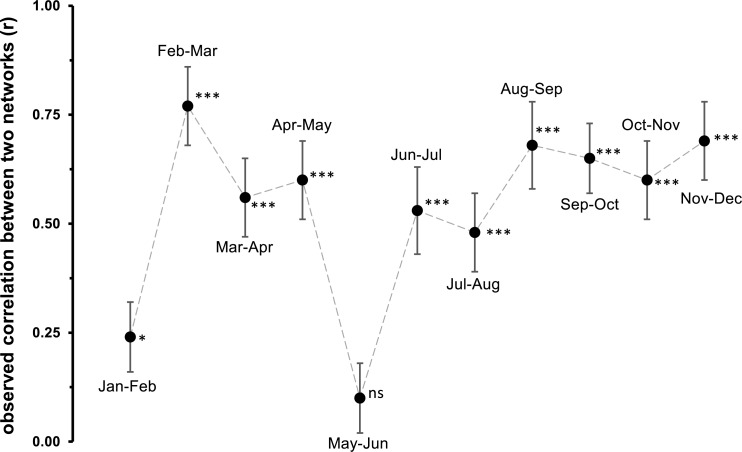
Observed correlation between two monthly networks. Jan, January; Feb, February; Mar, March; Apr, April; Jun, June; Jul, July; Aug, August; Sep, September; Oct, October; Nov, November; Dec, December. Symbols denote means, while error bar stands are the standard deviation (SD). The grey, dashed line illustrates the changed between months. Asterisks indicate different significant levels: *, *P* < 0.05, **, *P* < 0.01, ***, *P* < 0.001, ns, not significant.

Network analyses revealed changes in species distribution and specialization to the three microhabitats over the year (Table 1, Figs. 4A–4L). In general, the pooled bipartite network for all months (Fig. 4M) showed that the majority of oribatid mite species occurred in all three microhabitats (but in different frequencies) and thus was highly generalized (${H}_{2}^{{^{\prime}}}=0.156$). Focusing on individual microhabitats, however, oribatid mites showed higher generalization for moss (*d*′ = 0.076) as compared to dead wood (*d*′ = 0.134) and litter (*d*′ = 0.274). Comparison with null models revealed that this partitioning of mite communities across the microhabitats is a non-random distribution (all null models for every month: observed ${H}_{2}^{{^{\prime}}}\gg $ null model ${H}_{2}^{{^{\prime}}}$; *P* < 0.001). The monthly network structures were similarly partitioned as the pooled network (Fig. 4), yet ${H}_{2}^{{^{\prime}}}$ changed during the year (Table 2), but the community structures of consecutive months were highly correlated (Fig. 5). Communities of May and June were not correlated (*r* = 0.10; *P* > 0.05), resulting in a change of the bipartite network graph (i.e., moss in the central position; Fig. 4) based on *d*′ of the microhabitats (Table 2). Additionally, the overall microhabitat specificity ${H}_{2}^{{^{\prime}}}$ changed with air temperature (Table 2; Gaussian GLM: *F*_1,10_ = 11.34, *P* < 0.001), but not with air humidity (Gaussian GLM: *F*_1,10_ = 0.44, *P* = 0.51), indicating a more generalized distribution of oribatid mites at higher ambient air temperatures.

In addition, the standardized Kullback–Leibler divergence *d*′, representing the exclusiveness of the species found in a particular microhabitat per month, changed during the year (Table 2) and the three microhabitats differed significantly within each month (pairwise comparisons of ${H}_{2}^{{^{\prime}}}$-distances; all *P* < 0.01). Similar to the complementary specialization of the whole bipartite network (${H}_{2}^{{^{\prime}}}$), *d*′ values were lower in spring/summer (indicating a more generalized community in a given microhabitat) and higher in winter (indicating a more specialized community). Again, *d*′ values (Table 2) in microhabitats over the year were significantly influenced by air temperature (MANOVA: Wilk’s *λ* = 0.32, *F*_1,10_ = 5.58, *P* = 0.023), but not by relative air humidity (MANOVA: Wilk’s *λ* = 0.67, *F*_1,10_ = 1.29, *P* = 0.34). The effect of air temperature on *d*′ was driven by the changes of species exclusiveness in dead wood (univariate ANOVA: *F* = 18.72, *P* = 0.001) and moss (univariate ANOVA: *F* = 9.53, *P* = 0.012), while litter remained stable (univariate ANOVA: *F* = 1.61, *P* = 0.23).

**Figure 6:**
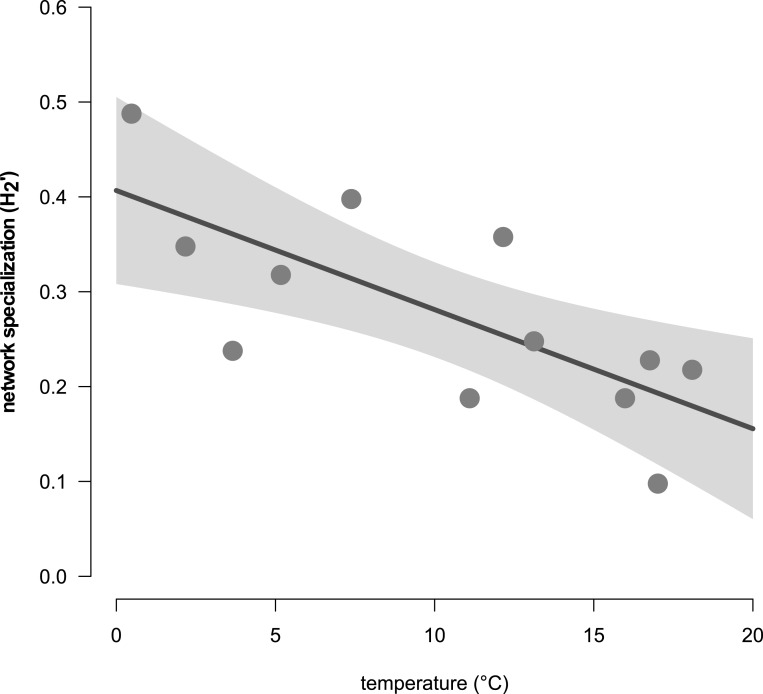
The relationship of the complementary network specialization (${H}_{2}^{{^{\prime}}}$) and the air temperature (in °C). Grey dots are ${H}_{2}^{{^{\prime}}}$ values in certain month; the grey area represents the 95% confidential interval. The dark grey lines is the linear regression curve of a Gaussian GLM (*F*_1,10_ = 11.34, *P* < 0.001).

**Figure 7:**
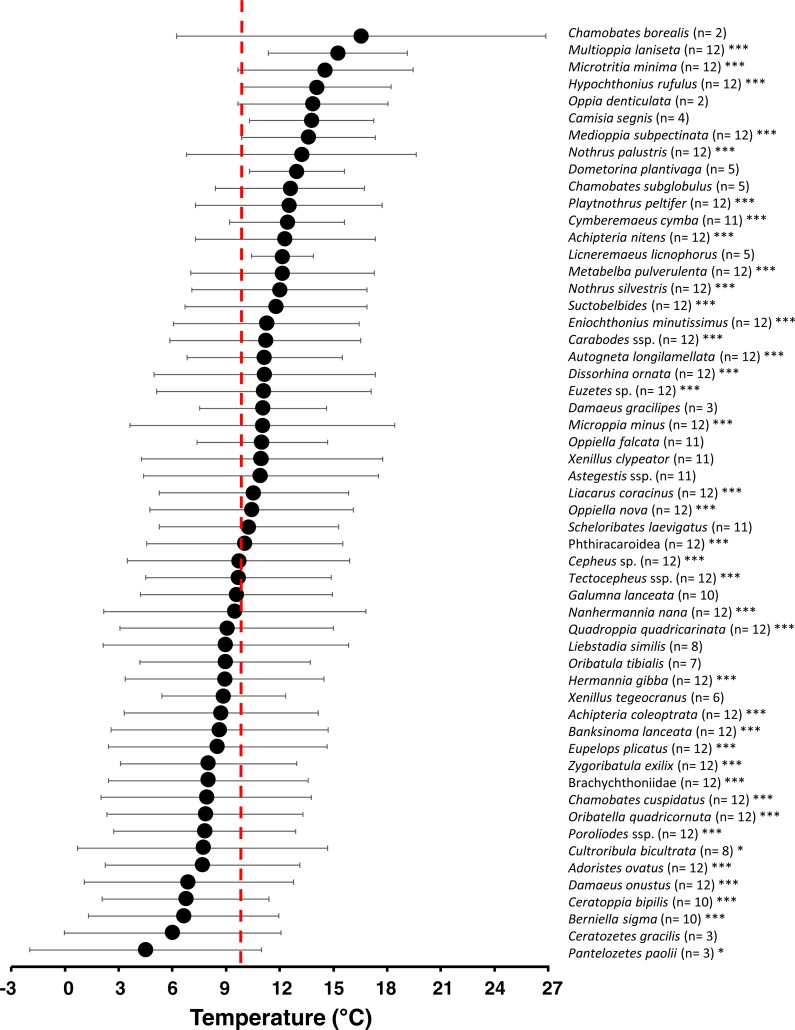
Temperature niche variation of 55 oribatid mite taxa. Numbers in brackets indicate the months of occurrence. Symbols denote means, while error bar stands are the standard deviation (SD). Red line indicates the annual mean air temperature. Asterisks indicate different significant levels: *, *P* < 0.05; **, *P* < 0.01; ***, *P* < 0.001.

The environmental occurrence niche analysis for air temperature *μ*_*i*_ (*ϑ*) revealed a generally broad temperature niche (=standard deviation of *μ*_*i*_, Fig. 6) for each oribatid mite species (5.3 ± 1.3 °C; mean ± SD). Comparisons with null models showed 39 out of 55 taxa had a non-random distribution across all months (and affiliated air temperatures), thus showing significant temperature niches *μ*_*i*_ (*ϑ*) (see asterisks in Fig. 7). While 35% of the species significantly occurred at higher air temperatures, 10% occurred in colder months, 25% reacted neutral with a *μ*_*i*_ (*ϑ*) near the mean annual air temperature (*ϑ* ≈ 10°C) and 30% showed no significant response (Fig. 7; Table S3). The environmental niche for relative humidity *μ*_*i*_ (RH) was also relatively broad for most oribatid mite species (12.0 ± 4.5%RH; mean ± SD), and according to null model comparisons 44 out of 55 taxa showed a non-random distribution related to relative air humidity across the year (see Fig. S1). Compared to air temperature, however, 60% of all species showed a significant neutral reaction to air humidity, while only 9% and 10% occurred at drier or wetter conditions, respectively, and 20% showed no significant response (see Table S4). Both environmental niches must be understood as “occurrence or distribution niche”, meaning that a certain oribatid mite species could most likely be found in the microhabitats investigated at a species-specific temperature or humidity range.

## Discussion

Most oribatid mite species were generally found in all three investigated microhabitats, but their distribution among them was unequal and fluctuated over the year. Moss had the highest individual abundance, but showed the strongest seasonal changes. When air temperatures were high, moss also had the highest species richness and diversity. Dead wood was characterized by a stable Shannon diversity indicating that this microhabitat is mainly inhabited by dead wood specialists that also occur in other microhabitats in low numbers. Litter was the most stable microhabitat over the year showing no influences of ambient air temperature and diversity only tended to increase with increasing air humidity.

All community parameters (abundance, species richness, diversity) confirmed former studies. The presence of a highly abundant oribatid mite community in mosses is well known (Aoki, 1967; Glime, 2013; Skubala, 2016; Wehner et al., 2016). Many oribatid mite species use mosses as microhabitat during their life cycle, for food or shelter, and associations can even be mutualistic (Cronberg, Natcheva & Hedlund, 2006; Glime, 2013). Dead wood is a special microhabitat that changes during its decomposition and provides special climatic conditions (Lachat et al., 2012). Mites associated with dead wood seem to increase the suitability of organic particles for decomposers (Norton, 1990) and contribute to both nutrient cycling and soil formation (Wallwork, 1979). Depending on the type of woody debris (e.g., coniferous or deciduous forests), reported densities and diversity values of dead wood-dwelling oribatid mite communities vary (Seastedt, Reddy & Cline, 1989; Skubala & Duras, 2008; Skubala, 2008). Additionally, the proportion of dead-wood specialists in such micro-communities has been controversial (see Skubala, 2008). While the proportion of specialists seems low in many studies and most dead-wood inhabitants can also be found on the forest floor (Seastedt, Reddy & Cline, 1989; Johnston & Crossley, 1996; Wehner et al., 2016), Skubala & Duras (2008) considered 63% of the species in downed logs to be specialists.

Although many studies on oribatid mites focus on the litter layer, this microhabitat is characterized by lower abundances compared to moss and dead wood. Nevertheless, litter provides a continuous refuge supplying food and protection for both litter specialists and those species retreating from microhabitats that are more disturbed (e.g., from moss patches if they are flooded or desiccated), indicated by constant abundances but fluctuating diversity parameters. Overall, our results confirmed the expectation that oribatid mite assemblage in moss, dead wood and litter differ in their community structure. Therefore, the variety of microhabitats contributes to the general animal species diversity in forest soil communities (Hammer, 1972; Wardle, 2002; Caruso, Toarmina & Migliorini, 2012; Bolger et al., 2014).

Although the distribution pattern and composition of oribatid mite communities are known from many studies, the mechanisms that affect these different community structures are more difficult to understand (Gergócs et al., 2011). Factors driving the distribution pattern of oribatid mite assemblages comprise niche dimensionality, resource portioning and resource quality, dispersal ability, local interactions and environmental filtering processes (Scheu & Drossel, 2007; Caruso, Toarmina & Migliorini, 2012; Maaßet al., 2015) and may affected by seasonality of communities. Our results indicate that seasonality of oribatid mite assemblages differ among microhabitats. Changes in abundances over the year were most pronounced in moss, probably due to strong alterations of the microhabitat structure during harsh environmental conditions such as drought or snow (Glime, 2013). Furthermore, changes in species richness and Shannon diversity may point to the usage of mosses as food resource by many different species. Seasonal changes of species abundances in litter were very low while fluctuations of diversity parameters were more pronounced, yet the exclusive specialization (*d*′) of oribatid mites towards this habitat also remained stable These results further emphasize the stability of the litter microhabitat and its function as an oribatid mite pool and transitional substrate for species dispersing to other microhabitats (“Connector-Hypothesis”; see Wehner et al., 2016).

In general, the composition of oribatid mite communities seems not to be seasonal but somehow related to temperature (Mitchell, 1977; Schenker, 1984; Stamou & Sgardelis, 1989; Webb et al., 1998; Irmler, 2006; Gergócs et al., 2011). Already, Schenker (1984) found species diversity to be higher at warmer temperatures. Irmler (2006) conducted a study in a beech forest in Germany over a period of seven years and observed no seasonality, but instead detected strong connection to annual mean air temperature. Similarly, Gergócs et al. (2011) found no seasonal change or recurring pattern of oribatid mite communities in a study over one and a half years in leaf litter and foerna substrates, but communities in moss were influenced by temperature (Gergócs et al., 2011).

Generally, the results of our study support the importance of ambient air temperature for structuring oribatid mite communities and again illustrate the need to investigate different microhabitats in order to obtain a complete picture of diversity at a forest site. However, differences in diversity are temperature-dependent rather than static. The complementary specialization of oribatid mite species towards different microhabitats changed from being slightly specialized at lower temperatures to more generalized (i.e., species are more equally distributed among litter, moss and dead wood) if air temperatures were high. While ${H}_{2}^{{^{\prime}}}$ followed this clear trend, the communities on which the networks based were highly correlated with each other. Only the months May and June were not correlated, because the exclusive specializations (*d*′) for moss and litter were much lower in June compared to May, yielding a lower overall ${H}_{2}^{{^{\prime}}}$. This again indicates a relatively high stability of microhabitat specialization of oribatid mite assemblages in consecutive months.

The increase of generalization at higher air temperatures is probably due to a broad environmental temperature occurrence niche of most oribatid mite species. However, while 30% did not react to air temperature, more species (35%) occurred at warmer air temperatures. For example, *Cymberemaeus cymba* and *Camisia segnis* occurred in moss, dead wood and litter only at air temperatures above the mean value of about 10 °C. Both species are known as typical inhabitants of tree bark (Behan-Pelletier & Walter, 2000; Erdmann et al., 2007) which is an insular microhabitat as compared to the continuous litter. Oribatid mite species living in these specialized, insular microhabitats must disperse more significant distances than litter species and the dispersal behavior that takes them into the litter ‘highway’, for example, may have some temperature threshold. If conditions are unfavorable for them to actively disperse, they ‘stay home’, i.e., the specialists enter a dispersal mode only at warmer air temperatures.

Furthermore, juveniles of highly specialized species are tightly bound to the microhabitat (e.g., burrowers in wood and lichen; Lebrun et al., 1991), while adults actively disperse only when development is complete. Additionally, the effect of temperature may be indirect via changing resource availability, the reproductive success or potentially predation pressure that forces oribatid mites to leave their favorite microhabitats. However, these assumptions need further investigation in future studies.

## Conclusions

Microhabitat specificity seems to increase at months with lower air temperatures. Most adult oribatid mite species have broad environmental temperature occurrence niches preferring higher temperatures. Therefore, if climatic conditions are unfavorable, species, especially those in specific, insular microhabitats, do not enter the dispersal mode but retreat to their specific terrain. Generally, seasonal changes in abundances are lowest in litter, intermediate in dead wood and highest in mosses, pointing to differences in microhabitat stability during seasons. On the other hand, seasonal changes of diversity parameters may be explained by dispersal dynamics of oribatid mite species among different microhabitats probably due to changing food conditions at warmer temperatures.

##  Supplemental Information

10.7717/peerj.4863/supp-1 Figure S1 Humidity niche of 55 oribatid mite taxa Numbers in brackets indicate the months of occurrence. Symbols denote means, while error bars stand are the standard deviation (SD). Red line indicates the annual mean air temperature. Asterisks indicate different significant levels: *, *P* < 0.05, **, *P* < 0.01; ***, *P* < 0.001.

10.7717/peerj.4863/supp-2 Table S1 Dry weights (gram) for ten replicates of dead wood, moss and litter from January to December 2016

10.7717/peerj.4863/supp-3 Table S2 Species abundances (individuals/kg dry weight) in the microhabitats moss, dead wood (dw) and litter from January to December 2016

10.7717/peerj.4863/supp-4 Table S3 Statistical results of the temperature occurrence niche analysis *μ*_*i*_(*ϑ*), environmental occurrence niche for air temperature; SD, standard deviation of *μ*_*i*_(*ϑ*).

10.7717/peerj.4863/supp-5 Table S4 Statistical results of the humidity occurrence niche analysis *μi* (RH), environmental occurrence niche for air humidity; SD, standard deviation of *μi* (RH).

## Additional Information and Declarations

### Data Availability

The following information was supplied regarding data availability: The raw data are included in the Supplemental Files.
