# Supplementary figures and images for: Seasonal fluctuation of oribatid mite communities in forest microhabitats
Source: PeerJ. 2018 Jun 4;6:e4863. doi: 10.7717/peerj.4863 (PMC5991301; doi:10.7717/peerj.4863)

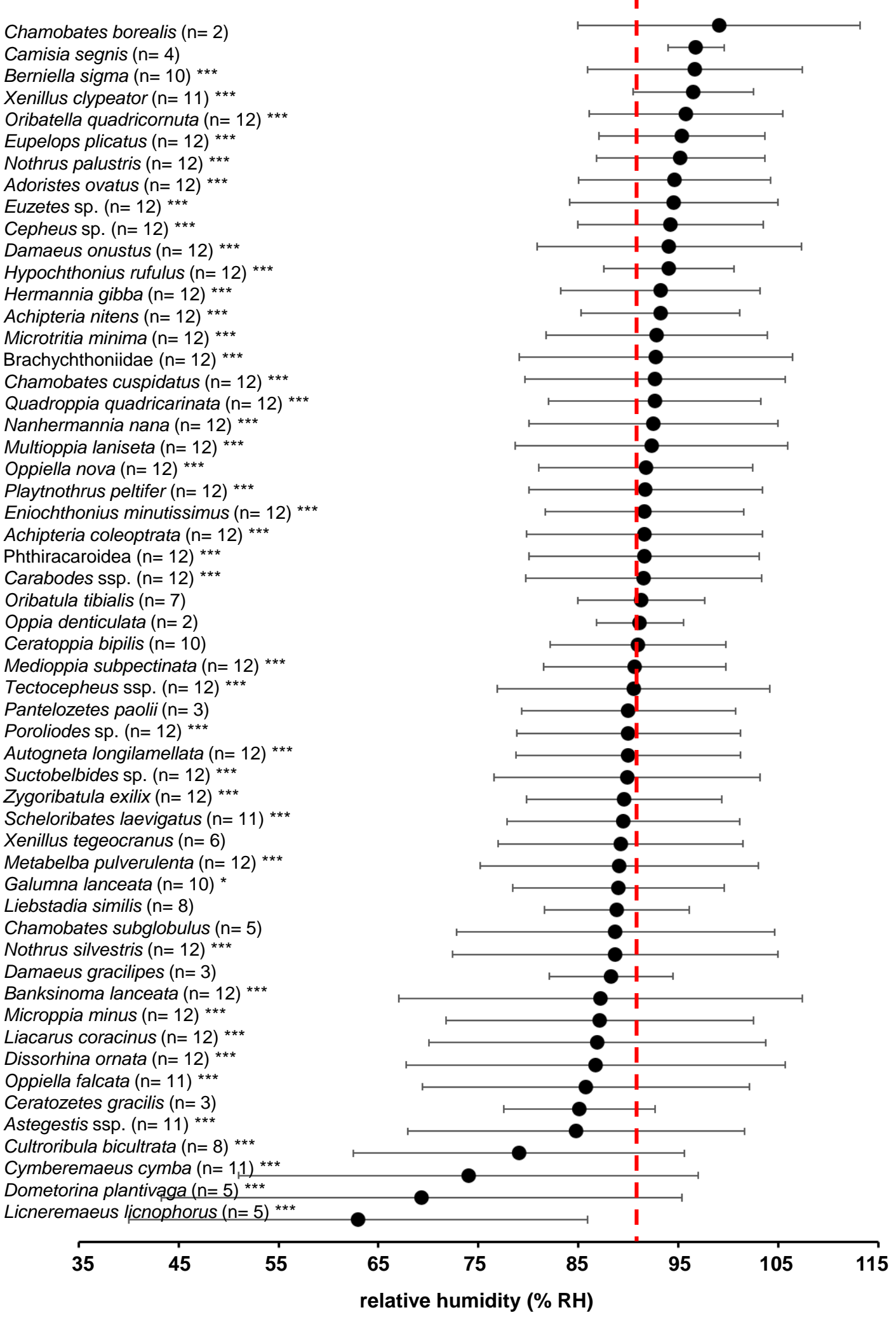

Supplement: Figure S1 — Numbers in brackets indicate the months of occurrence. Symbols denote means, while error bars stand are the standard deviation (SD). Red line indicates the annual mean air temperature. Asterisks indicate different significant levels: *, P < 0.05, **, P < 0.01; ***, P < 0.001. [file peerj-06-4863-s001.pdf]
